# Public health round-up

**DOI:** 10.2471/BLT.19.011219

**Published:** 2019-12-01

**Flooding in South Sudan**

Young people take shelter in a church in Panyagor, a town in Jonglei State, South Sudan, where an estimated one million people have been affected by flooding.

**Figure:**
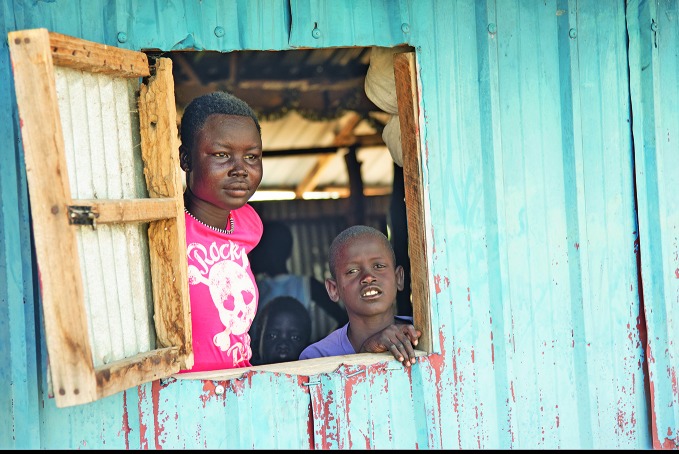
Helene Sandbu Ryeng/UNICEF

## Floods hit African and Eastern Mediterranean Regions

The World Health Organization (WHO) supported emergency response efforts, including delivery of supplies to populations impacted by severe flooding across the African and the Eastern Mediterranean Region in the first week of November.

As of 8 November, well over a million people had been affected in some of the worst-hit countries, including Benin, Cameroon, Central African Republic, Somalia, South Sudan and Yemen.

Floods can increase risk of illness or death from trauma, water-borne diseases and other infections that spread easily in overcrowded, temporary shelters.

WHO worked to reduce the risk of outbreaks of cholera, typhoid and other infectious diseases, in collaboration with governments of the countries concerned, other United Nations agencies and partners.

In South Sudan, where an estimated million people have been affected by flooding, WHO sent experts and airlifted medical supplies to support the government’s emergency response.

In Somalia, floods have led to the displacement of more than 300 000 people since September. As of 8 November, WHO had helped to deploy 30 emergency and rapid response teams and had distributed 483 medical supply packages.

In the Central African Republic, floods left 23 000 people displaced. WHO distributed mosquito nets and medical supplies.

In the Eastern Mediterranean Region, Cyclonic Storm Luban struck Yemen in October, killing 14 people and displacing 8000. As of 8 November, WHO had provided supplies and medical kits to support response efforts there.

https://www.who.int/news-room/detail/08-11-2019-who-aids-flood-hit-populations-across-africa-and-the-eastern-mediterranean-region

## Sudan cholera response

WHO staff began mapping areas of increased cholera risk in Khartoum State, Sudan, in support of efforts to prevent the spread of cholera in the country.

As of 3 November, Sudan’s Ministry of Health reported 332 suspected cases of cholera concentrated in Blue Nile and Sennar States. Two cases were confirmed in Khartoum State on 19 October.

Dr Naeema Al Gasseer, WHO Representative in Sudan, said that if the outbreak were not properly managed there could be serious consequences.

“More than eight million people live in Khartoum State, where the public health system is impacted by the economic crisis, recent flooding and ongoing outbreaks of infectious diseases,” she said.

The Sudan cholera outbreak was declared on 8 September. Some 1.6 million adults and children over the age of one year were vaccinated in Blue Nile and Sinnar states in mid-October. A second round of vaccinations was due to follow in November or in December.

http://www.emro.who.int/sdn/sudan-news/who-scales-up-cholera-vigilance-in-khartoum-sudan.html

## Ebola vaccine authorized

The European Commission (EC) decided last month to grant marketing authorization to the company Merck Sharp & Dohme B.V. for the Ebola virus vaccine, rVSV-ZEBOV-GP, which has proven to be effective and safe. As of 23 September 2019, over 223 000 people had received this vaccine during the ongoing Ebola virus disease outbreak in the Democratic Republic of the Congo.

To date, the vaccine has been provided to all people at high risk of Ebola infection including those who have been in contact with a person confirmed to have Ebola and all contacts of contacts.

The EC’s 11 November decision follows an 18 October announcement by the European Medicines Agency recommending conditional marketing authorization for the vaccine.

For the rVSV-ZEBOV-GP vaccine, African regulators joined the review process through a cooperative arrangement established by WHO, which aims to speed up the registration process for countries at risk of Ebola outbreaks.

https://ec.europa.eu/commission/presscorner/detail/en/ip_19_6246

https://www.who.int/news-room/detail/18-10-2019-major-milestone-for-who-supported-ebola-vaccine

## Ebola response health worker killed

A community health worker engaged in the Ebola response in the Democratic Republic of the Congo was killed in an attack in the Mandima Health Zone last month. The man’s wife was also critically injured.

WHO and partners condemned the attack, adding that acts of violence against individuals involved in the Ebola outbreak response are unacceptable and compromise response efforts.

As of 5 November, 3285 people were reported to have been infected with Ebola virus, 2191 of whom had died.

https://www.who.int/csr/don/07-november-2019-ebola-drc/en/

## Digital Health Group

The WHO Digital Health Technical Advisory Group met at WHO’s headquarters in Geneva for the first time on 24 and 25 October to develop a global strategy to accelerate the use of digital technologies in public health.

The group discussed data governance and ethical and equitable use of digital technologies. An objective of the group is the development of a global framework which WHO will use to validate, implement and scale up digital health technology. Advisory group members come from a wide range of fields, including artificial intelligence, virtual and augmented reality, biomedical innovation, robotic surgery, wearable technologies, health and wellness, ethics, governance, security, economics and law.

https://www.who.int/news-room/detail/25-10-2019-who-expert-panel-on-digital-health-meets-for-first-time

## Contraception cessation

Two-thirds of sexually active women who wish to delay or limit childbearing stop using contraception for fear of side effects and other health concerns and because they underestimate their chances of becoming pregnant, according to a 36-country study done by WHO.

The study, which was published on 23 October, explored the reasons for discontinuation of contraceptive methods used by women with a current unintended pregnancy.

The study found that unintended pregnancies ranged from 5% of all pregnancies in the Kyrgyz Republic to 60% in Colombia and Peru. In Central Asian and in six African countries, more than 80% of women with a current unintended pregnancy had not used any contraceptives in the previous five years.

More than 65% of women with an unintended pregnancy in the 36 low- and middle-income countries studied were either not using contraception or were using traditional methods, including withdrawal, periodic abstinence and the calendar rhythm method.

The study underlined the need for health systems to support use of suitable contraceptive methods, including long-acting methods, and to identify whether women have concerns about the methods they are using.

https://www.sciencedirect.com/science/article/pii/S0010782419304305?via%3Dihub

**Cover photo**

A flooded village in the Kurigram district of Bangladesh. Severe rainfall during the 2019 monsoon season has led to widespread flooding in Bangladesh, displacing thousands of people who have limited access to safe drinking water, food or sanitation.

**Figure:**
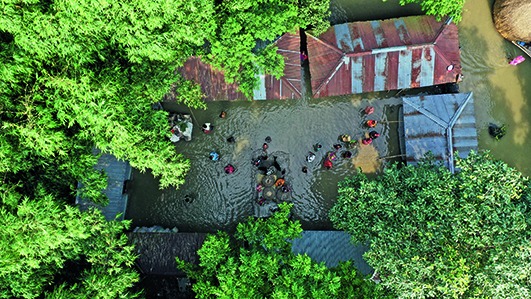
Credit: UNICEF/Zhantu Chakma

## Parliamentarians for universal health coverage

Members of the Inter-Parliamentary Union (IPU), adopted a resolution on achieving Universal Health Coverage (UHC) by 2030.

The 1800 members of the IPU met with UN and civil society partners in Belgrade, Serbia, from 13 to 17 October.

The resolution is designed to accelerate progress towards UHC and came one month after heads of state agreed a high-level United Nations Political Declaration on UHC in New York.

The resolution calls on WHO to work with the IPU and other partners to support the implementation of the resolution at the global, regional and country levels and to monitor progress.

“The resolution highlights the power of the collaboration between Inter-Parliamentary Union and WHO and builds on the memorandum of understanding we both signed in October 2018,” said Dr Tedros Adhanom Ghebreyesus, WHO Director-General.

He added: “Parliamentarians have a vital role to play in making this happen. Because it’s parliamentarians who pass laws and allocate funding. It’s parliaments who keep government accountable for the commitments it has made and who forge the partnerships that help countries make universal health coverage a reality.”

https://www.who.int/news-room/detail/17-10-2019-universal-health-coverage-passes-key-global-milestone

## Mexico validated rabies free

Mexico became the first country to receive WHO validation for eliminating dog-transmitted rabies as a public health problem. The achievement was announced on 11 November.

To achieve elimination, Mexico implemented a national strategy that included free, mass vaccination campaigns for dogs, continuous surveillance, public awareness-raising campaigns, timely diagnosis and post-exposure prophylaxis.

The country went from registering 60 cases of human rabies transmitted by dogs in 1990, to 3 cases in 1999, and zero cases in 2006. The last two cases occurred in two people from the State of Mexico, who were attacked in 2005 and presented symptoms in 2006.

Rabies causes 60 000deaths each year, mainly in Asia and Africa. In Latin America and the Caribbean, new cases of rabies were reduced by more than 95% in humans and 98% in dogs since 1983.

https://www.paho.org/hq/index.php?option=com_content&view=article&id=15585:mexico-is-free-from-human-rabies-transmitted-by-dogs&Itemid=1926&lang=en

**Looking ahead**

2 – 3 December. Global Vaccine Safety Summit, WHO headquarters, Geneva, Switzerland.

2 – 5 December. International Conference on AIDS and STIs for Africa, Kigali, Rwanda.

9 – 12 December. WHO global meeting to accelerate progress on SDG target 3.4. Muscat, Oman.

